# Autonomic neural control of motor activity in the intestine of freshwater barramundi (*Lates calcarifer)*

**DOI:** 10.1007/s00441-025-04034-5

**Published:** 2026-01-20

**Authors:** Hayley Rhodes, Lee Travis, Timothy J. Hibberd, Nick J. Spencer, James Harris

**Affiliations:** 1 Flinders University, Adelaide, SA Australia; 2 Visceral Neurophysiology Laboratory, College of Medicine and Public Health, Flinders University, Adelaide, SA Australia

**Keywords:** Enteric nervous system, Intestine, Peristalsis, Teleost fish, Myenteric plexus

## Abstract

Evolutionary pressure on the gastrointestinal (GI) tract of teleost fish differs substantially from those of terrestrial animals. The intestine is a pivotal organ in teleost, for maintaining osmotic balance against the surrounding aquatic environment; primarily through its role in water absorption. Intestinal sensory and motor functions of vertebrates are largely mediated by the enteric nervous system (ENS) embedded within the gut wall. Although the ENS has been described in several teleost species, the group comprised of more than 25,000 species displays remarkable ecological, anatomical, and physiological diversity. As such, species that display distinct adaptations, i.e., euryhalinity, may provide valuable comparative insights. Here we show the structure of the ENS within the intestine of barramundi (*Lates calcarifer*), a catadromous perch species with a unique life history and growing commercial relevance. Immunohistochemical labelling identified enteric neurons synthesising nitric oxide synthase (NOS) and calcitonin-gene-related peptide (CGRP), both of which were typically uniaxonal with smooth cell bodies. Qualitatively, these neuronal populations formed a weakly arranged enteric plexus, analogous to the myenteric plexus found in terrestrial animals. Quantitatively, the proportion of NOS immunoreactive neurons decreased along the rostro-caudal axis of the intestine without accompanying changes to the overall neuronal density. Video imaging of intestinal wall movements ex vivo identified multiple recurrent motility patterns which were hexamethonium-sensitive, suggesting regulation by nicotinic synaptic transmission within enteric pathways. Together, these findings show that enteric neurons are present in the barramundi intestine but form a comparatively less defined plexus than in higher terrestrial vertebrates. These enteric neurons are involved in the regulation of intestinal motility via nicotinic transmission.

## Introduction

The gastrointestinal (GI) tract is relatively unique among the internal organs of the vertebrate body as it contains its own intrinsic neural network, the enteric nervous system (ENS) (Furness and Stebbing 2018; Spencer and Hu 2020; Furness 2012). The ENS is a key regulator of GI motility, the mechanical process underlying the propulsion of content through the gut via smooth muscle contractions and relaxations (Spencer and Hu 2020; Furness 2012). The localisation of complete neuronal reflex pathways provides a degree of autonomy that allows the persistence of gut functions even if connection to the central nervous system is severed (Furness and Stebbing 2018; Gershon and Erde 1981; Olsson 2024). From an evolutionary perspective, an ENS has been identified in the intestine of all vertebrate and invertebrate species studied, including in those that lack a central nervous system such as Hydra (Furness and Stebbing 2018). Yet, most of our understanding of the ENS is derived from research in mammalian species and it contains a diverse population of neurons with different neurochemical and morphological characteristics that are foundational in comparative interpretations (Furness 2012, 2022; Spencer and Hu 2020). Like other biological systems, the structural complexity of the ENS appears to increase in more evolutionary derived vertebrates (Furness 2022; Furness and Stebbing 2018; Olsson and Holmgren 2011).

There are major functional differences in the role of the GI tract for water and ion absorption between terrestrial and aquatic vertebrates. Unlike terrestrial vertebrates, teleost fish utilise an anatomically simple GI tract to simultaneously comanage digestive and osmoregulatory functions across a range of environmental salinities (Ciavoni et al. 2024; Greenwell et al. 2003). Although the ENS has been described in several teleost species, the group which accounts for nearly half of all extant vertebrate species, is comparatively understudied and offers an abundance of opportunity to understanding the evolutionary and adaptive capacities of the ENS.

From studies in model species including zebrafish (*Danio rerio*), shorthorn sculpin (*Myoxocephalus scorpius*) and several salmonid species, we know that most enteric nerve cell bodies of the teleost ENS are solely contained within a myenteric plexus (Olsson 2009; Burnstock 1959). Instead of well-formed ganglia, neurons are considerably scattered, and the degree of which appears to vary between species (Olsson 2009). The nerve cell bodies occur at densities similar to those recorded in small mammals but differ markedly in size and morphology (Olsson and Holmgren 2011). Additionally, the submucosal plexus typically found in higher vertebrates is almost always absent in teleosts (Olsson 2009).

Barramundi (*Lates calcarifer*), also known as Asian seabass, is a large, euryhaline species of perch, and one of the few species of teleost that display a seasonal catadromous (freshwater to marine) migration and protandrous (male to female) transition later in life (Yue 2025). Barramundi have an important role in Australia’s aquacultural industry and have experienced a strong increase in commercial relevance in recent years (Grey 1987; Yue 2025). Their unique biology allows them to be cultivated in a wide range of salinities and within different production systems (Yue 2025). As such, detailed studies on the barramundi GI tract are readily available and provide a good understanding of the species’ anatomy, morphology, and general microbiota (Purushothaman et al. 2016; Zheng et al. 2019). These factors make barramundi an ideal model for studying the enteric nervous system in teleosts while diversifying from traditional teleost models.

Here we provide the first description of the ENS in the intestine of barramundi and characterise multiple distinct patterns of motor activity that can be autonomously generated ex vivo by the barramundi intestine. Ultimately, we aim to contribute to a foundational understanding of the GI tract’s neurobiology in teleosts.

## Materials and methods

Juvenile barramundi (*Lates calcarifer*), previously raised in freshwater (FW), were sourced from a local supplier (Hao Yi Live Seafood, South Australia, Australia). Individual fish were housed in 70L glass aquaria and acclimated for 1 week at 27 °C on a 12 h:12 h, light:dark period. Aquaria were housed within a FW aerated–recirculating system which constituted 3 aquaria supplied by a 50L sump (salinity 0–0.18ppt). Barramundi were fed a commercial pellet feed daily ad libitum for the duration of the experiment. This study, and all experimental procedures conducted within it, were performed in accordance with approval provided by the Flinders University Animal Ethics Committee for the project AEC BIOL6997-10.

### Sample collection

Individual fish were euthanised on the day of experimentation via the Iki-Jime method, consisting of a rapid spike to the brain. Descriptive measurements [total length (mm) and weight (g)] were collected promptly after euthanasia. A ventral incision was made from the vent to the peritoneal fins, exposing the abdominal cavity. The GI tract was dissected internally by making a blunt cut at the most orally accessible point of the oesophagus and at the anal pore; thus isolating the oesophagus, stomach, pyloric caeca, intestine, and rectum. The entire tract was removed and immediately placed into oxygenated Krebs–Ringer solution (140 mM NaCl, 2.5 mM KCl, 1 mM KH_2_PO_4_, 0.8 mM MgSO_4_, 1.5 mM CaCl_2_, 15 mM NaHCO_3_, 5 mM HEPES, and 10 mM D- Glucose). To isolate the intestine and remove the surrounding mesentery tissue, a finer dissection was performed under a stereo microscope (Olympus SZ61) in a petri dish containing carbonated (95% O_2_ and 5% CO_2_) Krebs–Ringer solution. A blunt cut was made immediately distal to the pyloric caeca and prior to the rectum to acquire the entire intestine as a single sample. If required, luminal contents were gently flushed out of the tract by pipetting Krebs–Ringer solution into the oral end of the intestine.

### Experimental set up

**Fig. 1:**
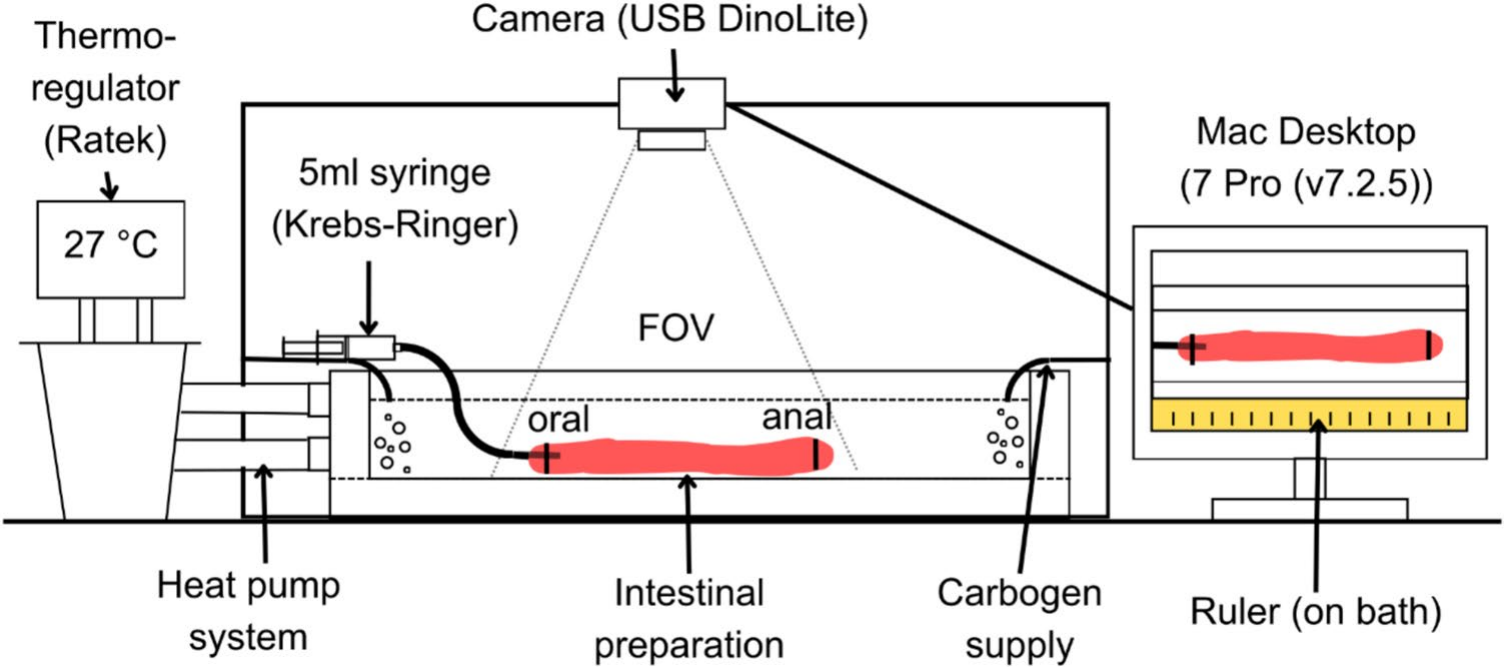
Experimental set up of an ex vivo motility study conducted on intestinal preparations of the barramundi (*Lates calcarifer*). Abbreviation - FOV, field of view

Intestinal preparations were mounted in a Slygard (Dow Corning)-lined organ bath (see Fig. 1 for a schematic diagram of the setup). The bath contained 250 mL of Krebs–Ringer solution that was heated and maintained at 26–27 °C via a thermoregulator and pump system. The oral end of the intestine was secured with thread to a 5 mL syringe containing Krebs–Ringer solution. The bath was continuously supplied with a steady flow of carbogen (95% O_2_, 5% CO_2_) at either end. A ruler was attached to the outer edge of the organ bath as a spatial reference in recordings, and a portable fluorescent light was positioned adjacent to the bath to optimise visual contrast between the intestine and the background. Videos were captured by a USB camera (Dino-Lite, model AM7515MZT, AnMo Electronics Corporation, Taiwan) connected to a Mac desktop computer running Lab Chart 7 Pro (v7.2.5; ADInstruments, Bella Vista, NSW, Australia). The resulting recordings had a resolution of 1280 × 960 pixels and consisted of 30–120 min recordings (9.15 frames per s).

### Spatiotemporal mapping

Intestinal motility was visualised and quantified using a protocol adapted previously from the laboratory (Costa et al. 2019). Briefly, overhead video recordings of intestinal preparations were transformed into maps of circumferential gut diameter (diameter maps; Dmaps) with the spatiotemporal mapping technique described by Hennig et al. (1999) using a custom-made edge detection program in Matlab (MathWorks, Inc., USA). Regions of minimal diameter (i.e., contraction) are represented as lightest pixels and maximal diameter (i.e., relaxation/accommodation) is represented by darkest pixels. As no previous literature exists on motility patterns in the barramundi, intestinal motility patterns were characterised as any repeated muscle contractions that were visually distinct and identifiable in both the video recordings and subsequently produced Dmaps. The frequency (contractions per min), velocity (mm per s), distance (% of IL), and direction (antegrade or retrograde) of these contractions were manually extracted from the Dmaps in a custom-written software (PlotHRM).

### Motility experiment design

Experiments began with a 45 min acclimation period, followed by a two-hour study. The two-hour recording periods were divided into four consecutive 30 min subperiods: control, distention, hexamethonium (300 µM), and tetrodotoxin (1 µM). Following the initial 30 min control period, the anal end of the intestine was cannulated with thread and distended with Krebs–Ringer solution from the oral end. Krebs infusion volumes varied (0.5–2 mL), relative to the intestine’s final length. During the third 30 min period, the nicotinic receptor antagonist, hexamethonium (HEX, 300 µM bath concentration) was applied to the distended intestine, and 30 min later the sodium channel blocker tetrodotoxin (TTX; 1 µM bath concentration) was cumulatively applied for the final 30 min period.

### Histology and immunohistochemistry

Upon the completion of each motility study, whole intestinal preparations were transferred into a petri dish containing phosphate buffered saline (PBS). The organ was then either (1) maintained as a tubular sample for histology or (2) cut longitudinally along the mesenteric border and pinned flat muscle side uppermost to a Sylgard (Dow Corning)-lined petri dish for wholemount immunohistochemical staining. Both forms of tissue were fixed in 4% paraformaldehyde (PFA) overnight at room temperature. Following fixation, samples were rinsed with PBS and stored at 4 °C.

From fixed tubular samples, 3 segments (~ 0.5 mm in width) were cut, representing the proximal, mid, and distal intestine. These segments were placed into cassettes and loaded into an automated tissue processor (HistoCore PEARL, Leica Microsystems), where they were processed overnight. After processing, 9 × 5 µm thick cross sections were embedded, cut, and stained with hematoxylin and eosin (H&E). The resulting slides were imaged using an Olympus VS200 Slide scanner at 20 × and 40 ×.

Immunohistochemical labelling was conducted on flat tissue samples. Four intestinal segments (3 cm sections) were cut from the proximal (2 ×), mid, and distal intestine. Before staining, the mucosa was stripped from each segment with fine forceps. Segments were pretreated with 3 × 10 min immersions in dimethyl sulfoxide (DMSO) and rinsed with 3 × 10 min washes in PBS. They were then immersed in a 10% normal horse serum (NHS) (Life Technologies Gibco #16,050–122) solution with antibody dilutant on a shaking platform for 1 h. Three segments, one representing each region, were transferred into a primary antibody solution for 5 days. The fourth segment, a negative-only control, was left in 10% NHS for the subsequent time. All four segments were then rinsed in 3 × 10 min washes of PBS before immersion in secondary antibody solution for 2–3 days on a shaking platform, shielded from light exposure. The stained segments were then rinsed with 3 × 10 washes in PBS and then equilibrated in a series of carbonate-buffered glycerol solutions (50%, 70%, and 100% solutions; 3 × 10 min) before mounting on glass slides in buffered glycerol (pH 8.6). Preparations were imaged on an Olympus IX71 Inverted Fluorescence Microscope using 3 channels, FITC (Excitation (Ex): 490 nm; Emission (Em): 525 nm), Cy3 (Ex: 554 nm; Em: 568 nm), and Cy5 (Ex: 647 nm; Em: 666 nm).

### Antibody characterisation

The primary and secondary antibodies used, and their respective fluorochromes are provided in Table 1.

**Table 1:** Primary and secondary antisera and their dilutions used in immunohistochemical investigations of the ENS in the barramundi (*Lates calcarifer*)

Antibody	Host	Dilution	Supplier	Cat#	RRID
*Primary antisera*					
Calcitonin gene-related peptide (CGRP)	**Rabbit**	**1:2000**	**Bachem**	**T-4032**	**AB_518147**
HuC/D	**Mouse**	**1:100**	**Invitrogen**	**A21271**	**AB_221448**
Nitric Oxide Synthase (NOS)	**Sheep**	**1:2000**	**Emson**	**K205**	**AB_2314957**
*Secondary antisera*					
Anti Rabbit IgG – Cy3	**Donkey**	**1:200**	**Jackson**	**711,165,152**	**AB_2307443**
Anti Sheep IgG – Cy5	**Donkey**	**1:200**	**Jackson**	**713,175,147**	**AB_2340730**
Anti Mouse IgG – FITC	**Donkey**	**1:100**	**Jackson**	**85,319**	**AB_2340792**

### Calcitonin gene-related peptide

The calcitonin gene-related peptide (CGRP) polyclonal antibody (T-4032, Bachem; previously IHC6006, Peninsula) was raised in rabbit against a rat αCGRP peptide. Western blots performed on horse ileum reveal a single band at 14 kDa, consistent with the molecular weight of CGRP (Russo et al. 2010).

### Hu C/D

The monoclonal HuC/D antibody (A21271, Invitrogen) was raised in mouse against a human HuD peptide conjugated to a keyhole limpet hemocyanin. Western blots performed on rat brain generated bands at 36, 40, and 42 kD (Pascale et al. 2004). This antibody is a common pan neuronal marker applied to studying the enteric nervous system in vertebrates and has been successfully applied on multiple occasions in teleosts (Olsson 2011a, b; Li and Furness 1993).

### Nitric oxide synthase

The neuronal nitric oxide synthase (NOS) polyclonal antibody was raised in sheep against recombinant rat brain neuronal NOS. Western blots performed on inferior mesenteric ganglion and pelvic ganglia in guinea pigs generated an intense band at 160 kDa (Olsson et al. 2006), consistent with its predicted molecular weight (Boissel et al. 1998).

Secondary-only controls were run for each series of samples and showed no non-specific staining. Additional antibodies were tested: including choline acetyltransferase antibody (ChAT; P3YE, Schemann), calbindin (CB38a, Swant), and calretinin (CR7697, Swant), but these failed to return a positive response.

### Image analysis

All measurements on acquired images (video stills, microscope slides) were performed in Qupath 0.5.1 with the Fiji software extension Image J and exported into Excel. Micrographs of neuron immunolabelling were additionally colourised and merged in Krita 5.1.3.

The width of the intestine was measured manually and in total, 25 measurements were taken per region for 28 fish sampled. The width of the circular and longitudinal muscle was taken in a similar manner, from the H&E-stained slides. Between 200 and 300 measurements were taken per muscle layer in each region and 3 fish were sampled.

To measure soma size, neuron density, and proportion, composite images of mucosal-free intestinal segments, stained with the pan neuronal marker HuC/D and nitric oxide synthase (NOS) (Table 1), were taken at 10 × and 20 × objectives and analysed in QuPath. Between five and eight images were analysed per intestinal region, and 3 fish were examined. The outline of complete and unobstructed nerve cell bodies, showing positive immunoreactivity to NOS, was manually drawn using the freehand brush tool while only observing the NOS-positive channel of the composite image. The Hu-positive channel was overlayed, and cell bodies immunoreactive to the pan-neuronal marker Hu, but not nitric oxide synthase, were measured separately. To better reflect neuron density, the raw counts of neurons immunoreactive to Hu were normalised to the sampled area (neurons per mm^2^). This normalisation accounted for variations in sample area to allow for a more accurate comparison of neuronal density across different regions.

### Statistical analysis: morphology

Qualitative data was formatted in Excel prior to analysis. Statistical analysis was performed in R4.4.1, within RStudio 2024.04.2. For frequentists approaches, a significance level was set at 0.05. For Bayesian approaches, results were considered meaningful in the 95% credible intervals (CIs) that did not cross 0.

Differences in tissue thickness and intestinal width between regions (proximal, mid, distal) were analysed using non-parametric methods (Friedman Test) as the data violated normality assumptions (Shapiro Wilk *p* < 0.05) and could not be appropriately transformed. As such, summary data are presented as median ± interquartile ranges (IQR). When satisfied, a Wilcoxon signed-rank test was used for post-hoc pairwise comparisons to identify differences between specific pairings of salinities.

To assess differences in soma size, enteric neuron distribution, and the proportion of nitrergic neurons across different regions, normality for each set of data against region was tested using the Shapiro–Wilk test, and homogeneity of variances across groups was assessed using Levene’s test. When the data met the assumptions of normality and equal variances between intestinal regions (proximal, mid, distal), parametric testing was used. A one-way ANOVA was performed to compare the mean neuron densities or the mean nitrergic proportions of the three regions. When satisfied, a Tukey’s HSD post hoc test would be applied to identify significant differences between pairs of regions. As such, the summary data for neuronal density and nitrergic proportions are presented as means with standard errors.

### Statistical analysis: intestinal motility

The distribution of raw motility data (frequency, velocity, and direction) across freshwater acclimated barramundi (*n* = 15) always displayed a strong positive skew and violated the assumptions of normality from the applied Shapiro–Wilk test (*p* < 0.05). These violations could not be resolved through transformation. The structure of the data, which contained repeated measures, the random effect of individual fish variability, and several covariates (fed status, bath temperature, time in experiment, RIL), supported the use of a Bayesian approach. Therefore, differences in separate intestinal motility parameters were analysed using Bayesian generalised linear mixed models (GLMMs), implemented via the ‘brms’ package in R 4.4.1.

Distributions for each parameter were selected based on their respective nature: gamma hurdle models were used to fit frequency and velocity, due to the presence of biologically relevant zeros and their positive skew. A Bernoulli distribution was used for direction, which was a binary outcome, and a beta distribution for distance, formatted as a proportion bounded between 0 and 1. As there is no relevant information to aid in the establishment of priors, the default ones fitted by ‘brms’ were used consistently.

The model selection process involved a series of model comparisons using LOOIC (leave-one-out information criteria) from the ‘loo()’ function part of the ‘stan’ package in R. Following the principle of parsimony, models were built and tested in order of simplicity. Starting from the fixed effects (Treatment) and random effects (Fish ID), complexity was increased through the addition and combination of co-variates. None of the tested covariates alone improved the model fit against its simplest form, which was supported by a lower value of LOOIC for the simplest model. Additionally, combinations of covariates resulted in an unsustainable model that consistently experienced convergence issues. As such, the final model implemented was simply:

$$\mathrm{Parameter}\;\sim\;\mathrm{Treatment}\;+\;(1\left|\mathrm{Fish}\right.\;\mathrm{ID})$$

This model with the appropriate distribution was fitted for parameters: frequency, velocity, direction, and distance travelled. After running the models, posterior predictive checks were completed using visual assessment of scatter-style predictive plots. Heteroscedasticity was assessed by the lack of clear patterns and a consistent spread. These checks indicated good agreement between observed and predicted values, with no strong evidence of systematic bias or poor fit.

Posterior distributions of model parameters were summarised using the posterior means and 95% credible intervals (CIs). Visualisations of treatment effects were generated using estimated marginal means (EMMs) extracted using the ‘emmeans’ package. These represented model adjusted estimates that provided clearer visual comparisons of the treatment effect alone, while posterior means and 95% CIs from the full model output, that better indicate the additional random effect, were used to formally report treatment effects.

## Results

**Fig. 2:**
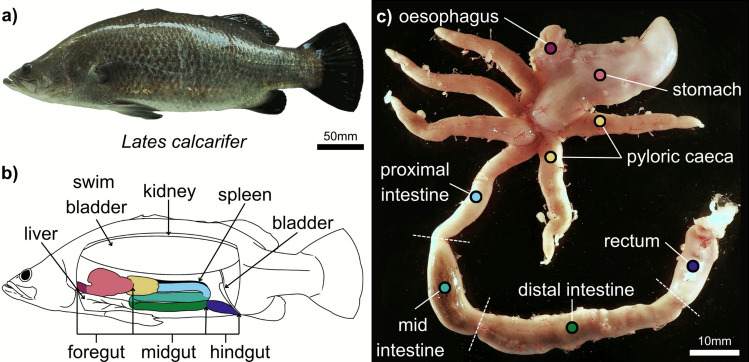
Gross anatomy of the isolated barramundi GI tract. **a** Photograph of a barramundi sampled within this study. **b** A simplified schematic diagram showing the orientation of the GI tract (coloured) and associated organs within the body. **c** Representative example of a dissected barramundi GI tract used within this study. The sampled fish had recently been fed prior to euthanasia, so digestive contents within the mid and distal intestine can be observed. Coloured circles in **c** correspond to the same-coloured regions of the GI tract in **b**

The anatomy of the barramundi GI tract followed a general teleost structure (Fig. 2 a - c). The foregut consisted of a short, thick oesophagus that extended directly into a y-shaped stomach (Fig. 2c). The stomach comprised thick, muscular tissue that funnelled into the pylorus, a muscular sphincter that controls the passage of food from the stomach to the intestines, at the posterior end marking the end of the foregut.

The midgut comprised the pyloric caeca; finger-like projections that extend from the pylorus in some teleost species, and the intestine. The barramundi possessed five well-developed pyloric caeca that were oriented caudally in the body and covered in a thick layer of mesenteric tissue. Notably, the pyloric caeca displayed unique movements visible to the naked eye during dissection, suggesting that individual caeca can generate motility ex vivo.

The intestine presented as a uniform tube that extended from the anal end of the pylorus to the rectum, lacking more compartmentalised regions typically observed in higher vertebrates (e.g. small and large intestines). The relative intestinal length (RIL) of the sample barramundi was 0.41 (SD: 0.06), translating to 41% ± 6% of the species’ total body length.

During dissection, it was evident that the intestine had a rich vascular supply, visible by the prominent network of blood vessels within a dense coating of mesentery tissue. Two flexures held the intestine in an s-shaped configuration. These flexures were useful to distinguish the three intestinal regions, which became visually homogenous once the mesentery was removed. Various names exist for these regions in the literature; in this study, they will be referred to as the proximal, mid, and distal intestine.

The proximal intestine was positioned immediately caudal to the base of the pylorus and was relatively narrow in diameter compared to the proceeding intestinal regions. The tissue appeared thick and opaque, even in a distended state (i.e. when digestive content was present) (Fig. 2c). The mid intestine maintained a narrow diameter, but the tissue appeared more translucent and less uniform in width. In addition to the flexure, the transitionary point from the mid- to the distal intestine was identifiable by a rapid and marked increase in diameter. The tissue of the distal intestine was visually much thinner, relative to the preceding regions. As such, digestive content, when present, was readily visible through the muscularis wall (Fig. 2c).

Consistent with visual assessment, the intestinal width (nondistended diameter) significantly differed between regions (*n* = 15, Friedman: *p* = 0.043). The distal region had the greatest diameter, with a median average of 3.83 mm (IQR: 3.32–4.74 mm). The proximal and mid regions were narrower and similar in width, with median averages of 2.85 mm (IQR: 2.48–3.39 mm) and 2.89 mm (IQR: 2.62 mm), respectively. Post hoc tests revealed a significant difference in width between the mid and distal region (Wilcoxon: *p* = 0.012) but not between the distal and proximal regions (Wilcoxon: *p* = 0.059). Additionally, there was no significant difference between the proximal and mid regions (Wilcoxon: *p* = 1.000).

The hindgut consisted only of the rectum, separated from the intestine by a prominent sphincter. The rectum was bulbous in shape and comprised of dense and opaque muscle tissue (Fig. 2c).

### Histology

**Fig. 3:**
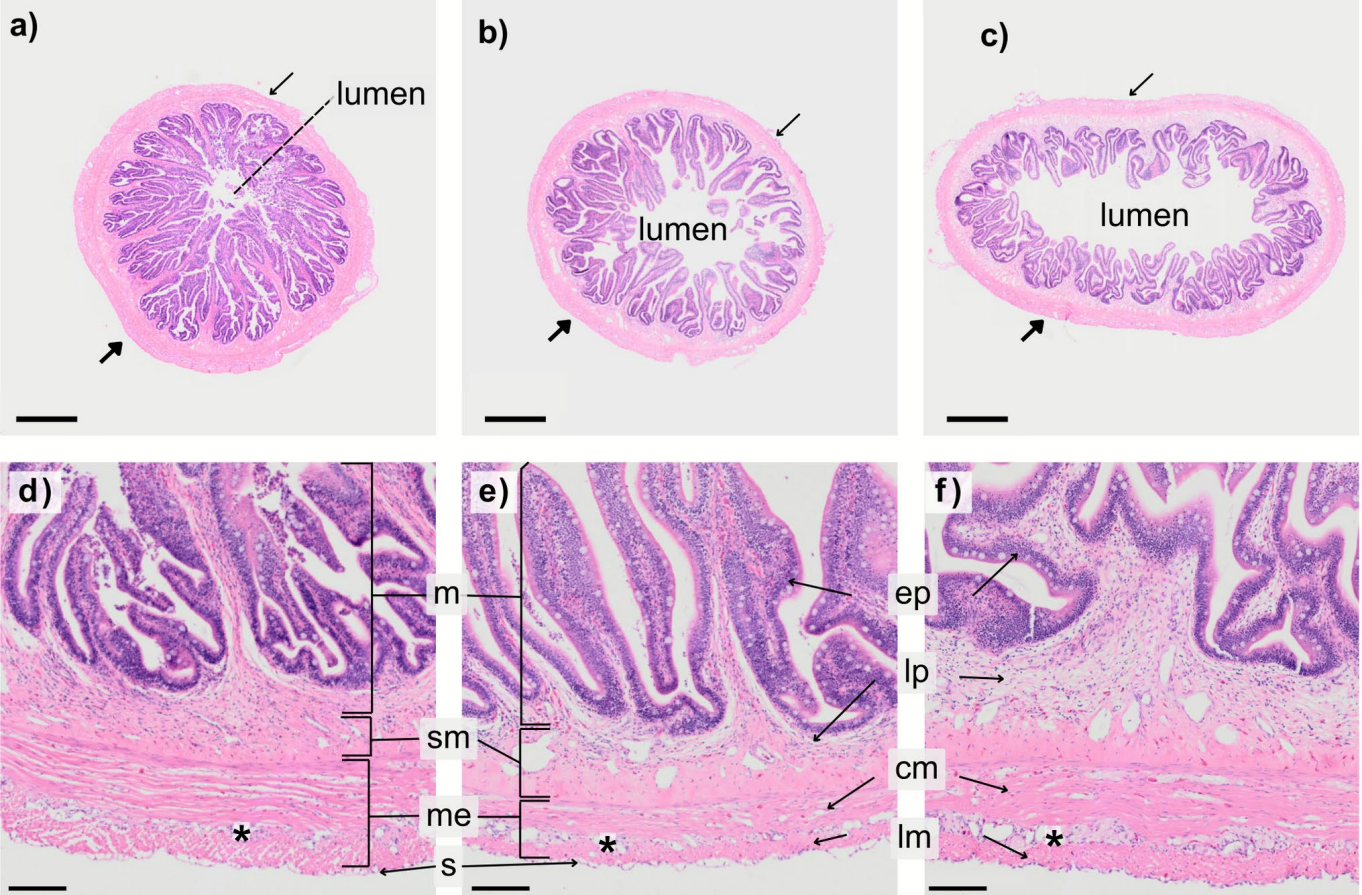
Transverse sections of the barramundi (*Lates calcarifer*) intestine, stained with hematoxylin and eosin and captured at 20 × magnification. **a**, **d** The proximal region. **b**, **e** The mid region. **c**, **f** The distal region. Widening of the lumen is visually evident as the intestine progresses. The walls of the intestine are arranged into 4 layers; the mucosa (m), submucosa (sb), muscularis externa (me), and the serosa (s). Within these layers are different tissue types including epithelium (ep), a lamina propria (lp), and both circular (cm) and longitudinal (lm) smooth muscle tissue. Thin arrows (a–c) indicate a thinning of the muscularis externa around the circumference of the intestine (mesenteric side), whereas thicker arrows indicate a thickening. The asterisk symbol (*) labelled between the two-muscle layers in each region indicates possible visual evidence of a myenteric plexus. Scale bar (**a**–**c**) = 800 µm, (**d**–**f**) = 100 µm

Histological examination of hematoxylin and eosin (H&E) stained sections of barramundi intestine (*n* = 3) revealed four distinct tissue layers: the mucosa, submucosa, muscularis externa, and the serosa (Fig. 3). The mucosa consisted of an epithelium and lamina propria. The epithelium was lined with columnar cells and folded into villi that show regional differences in structure and abundance (Fig. 3). In the proximal intestine, the villi were long and branching, with prominent crypts (Fig. 3). Compared to the preceding regions, the lumen was small. The number of villi ranged from 13 to 17 per section throughout the proximal and mid intestine, but in the latter, the height of the villi drastically reduced, and the lumen widened. The villi no longer branched, but crypts were still present. In the distal intestine, the lumen widened considerably; the villi were short and thick, but densely folded and abundant, with the number ranging from 19 to 25 per section (Fig. 3).

The lamina propria, a thin region of highly vascularised connective tissue, was comparably dense in the proximal intestine. It reduced in density but increased in width as the GI tract progressed. The barramundi, like other teleosts, lacked a muscularis mucosa, which separates the lamina propria from the submucosa in higher vertebrates. The submucosa consists of a dense collagen-rich and fibrous tissue that extends from the lamina propria to the muscularis externa. No evidence of a submucosal plexus, or submucosal neurons was identified with light microscopy.

The muscularis externa was composed of two layers of smooth muscle tissue that were oriented perpendicular to one another. The inner circular layer was well established, whereas the outer longitudinal layer was discontinuous around the circumference of the gut, featuring a distinct thinning at the mesenteric attachment. The thickness of both muscle layers varied around the circumference of the intestine in tandem (Fig. 3a–c). On few occasions, a complete absence of longitudinal muscle on the mesenteric side coincided with a thickening of the circular muscle that extended to the outer perimeter of the intestine. Despite these local structural variations, the average thicknesses of each muscle layer did not significantly differ between regions of intestine (Friedman, *p* > 0.05). In higher vertebrates, the myenteric plexus comprises the second major structure of the ENS and is located between the circular and longitudinal layers of the muscularis externa. In the barramundi intestine, a continuous layer of connective tissue was identified between such two layers (Fig. 3).

### The Barramundi ENS

**Fig. 4:**
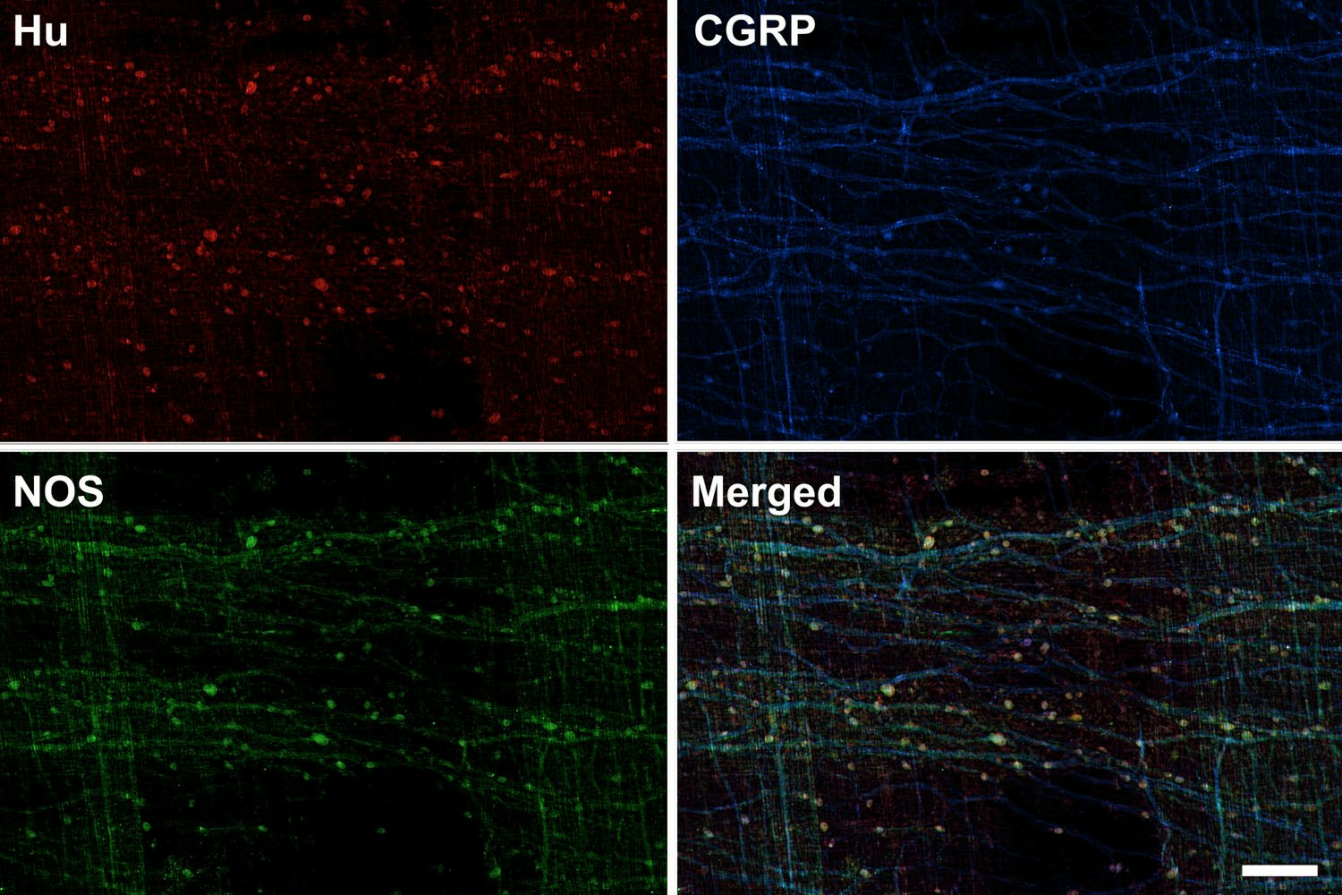
Matched micrographs of immunoreactivity to antibodies for Hu, CGRP, and NOS in the proximal intestine of the barramundi (*Lates calcarifer*), imaged at 4 × magnification. Enteric neurons (immunoreactive to Hu) were distributed evenly between the two layers of smooth muscle tissue. Neurons are connected by nerve fibres positive for CGRP and NOS. Note the preferential longitudinal (horizontal) orientation of nerve fibres in the plexus, with bands of circumferential nerve fibre bundles in overlying circular muscle (running in the vertical axis). Here, the absence of circumferentially oriented nerves near the centre of the field of view is due to the removal of a strip of circular muscle by sharp dissection, confirming the location of circumferential nerve fibres in the circular muscle layer. Scale bar = 200 µm

All wholemount preparations from every intestinal region, tested with antisera for the pan-neuronal marker Hu, exhibited positive immunoreactivity (*n* = 9). This labelling revealed numerous structures consistent with cytoplasmic labelling of nerve cell bodies (Fig. 4). All the observed Hu-positive nerve cell bodies were situated between the two layers of the muscularis externa. As none could be identified in the submucosa, it was evident that barramundi possessed a myenteric, but not submucosal, plexus.

Co-immunolabelling Hu with antisera for the neuropeptide calcitonin gene-related peptide (CGRP), and for the inhibitory transmitter nitric oxide synthase (NOS), revealed an interconnected network of nerve fibres. Numerous Hu-labelled nerve cell bodies also contained NOS immunoreactivity. CGRP only labelled nerve fibres (Fig. 4). At the level of the myenteric plexus, nerve fibres preferentially oriented in the longitudinal direction, whilst in the circular muscle, numerous bundles of varicose nerve fibres ran parallel to the circumferentially oriented muscle cells.

In total, 3335 labelled neurons were analysed across all regions (998–1277 per region) from the three fish sampled. Most neurons appeared as isolated cells, with occasional small clusters of 3–6 neurons. The overall distribution was relatively sparse (i.e. not obviously ganglionated) and consistent along the intestine. Quantification of Hu-positive neurons, normalised per mm^2^, showed no significant differences in neuron density across the three regions (one-way ANOVA, *p* > 0.05).

**Fig. 5:**
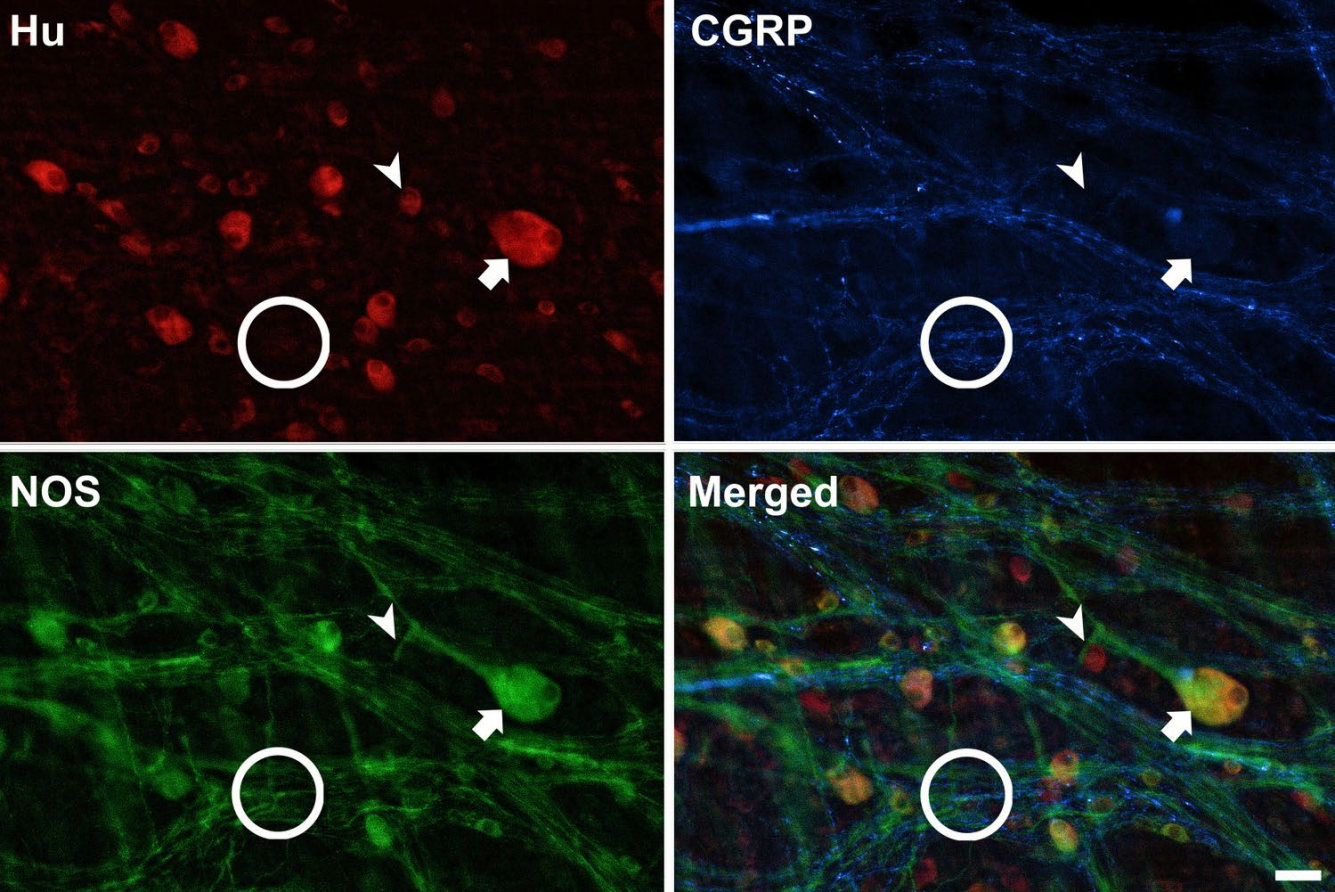
Matched micrographs of Hu, CGRP, and NOS immunolabelling in the proximal intestine of the barramundi (Lates calcarifer), imaged at 20 × magnification. Nitrergic neurons showed immunoreactivity to both Hu and NOS (arrows), whereas non-nitrergic neurons showed immunoreactivity to Hu only (arrowhead). Nerve fibres positive for CGRP travelled along similar pathways to NOS-containing fibres but did not consistently colocalise (circled areas). Scale bar = 50 µm

The morphology of barramundi enteric neurons featured a smoothly contoured soma, characteristic of Dogiel II morphology. No multiaxonal enteric neurons were positively identified, but single axons were observed bifurcating close to their cell body (see Fig. 5).

Nitrergic neurons were identified by their positive co-localisation of NOS and Hu (Fig. 5). On rare occasions (2 neurons), NOS-immunoreactive nerve cell bodies were identified that lacked a detectable positive reaction to Hu. The proportion of nitrergic neurons ranged from 51.5% (SE: 1.5%) in the proximal intestine to 50.4% (SE: 1.6%) in the mid-intestine, and 43.9% (SE: 1.3%) in the distal region. There were no significant differences in the proportions of nitrergic neurons between regions (one-way ANOVA, *p* > 0.05).

The density of myenteric neurons, quantified as the number of cells, normalised per area (cm^2^), decreased distally along the intestine. The lowest densities were observed in the distal intestine [28 469 ± 14 670 cells per cm^2^, *n* = 1314], followed by the mid [34 646 ± 15 135, *n* = 887], then proximal [39 098 ± 18 862 cells per cm^2^, *n* = 1134] intestine. There were no significant differences in density between regions (one way ANOVA, *p* > 0.05). The density of nitrergic neurons followed the same trend, where the lowest density was observed in the distal intestine [10 872 ± 333 cells per cm^2^, *n* = 643]. This was significantly lower than the mid [17 007 ± 8 644 cells per cm^2^, *n* = 527] intestine (Tukey HSD, *p* = 0.047) and the proximal intestine [16 538 ± 7 069 cells per cm^2^, *n* = 584] (Tukey HSD, *p* = 0.036). There were no significant differences in neuron density between the mid and proximal intestine.

**Fig. 6:**
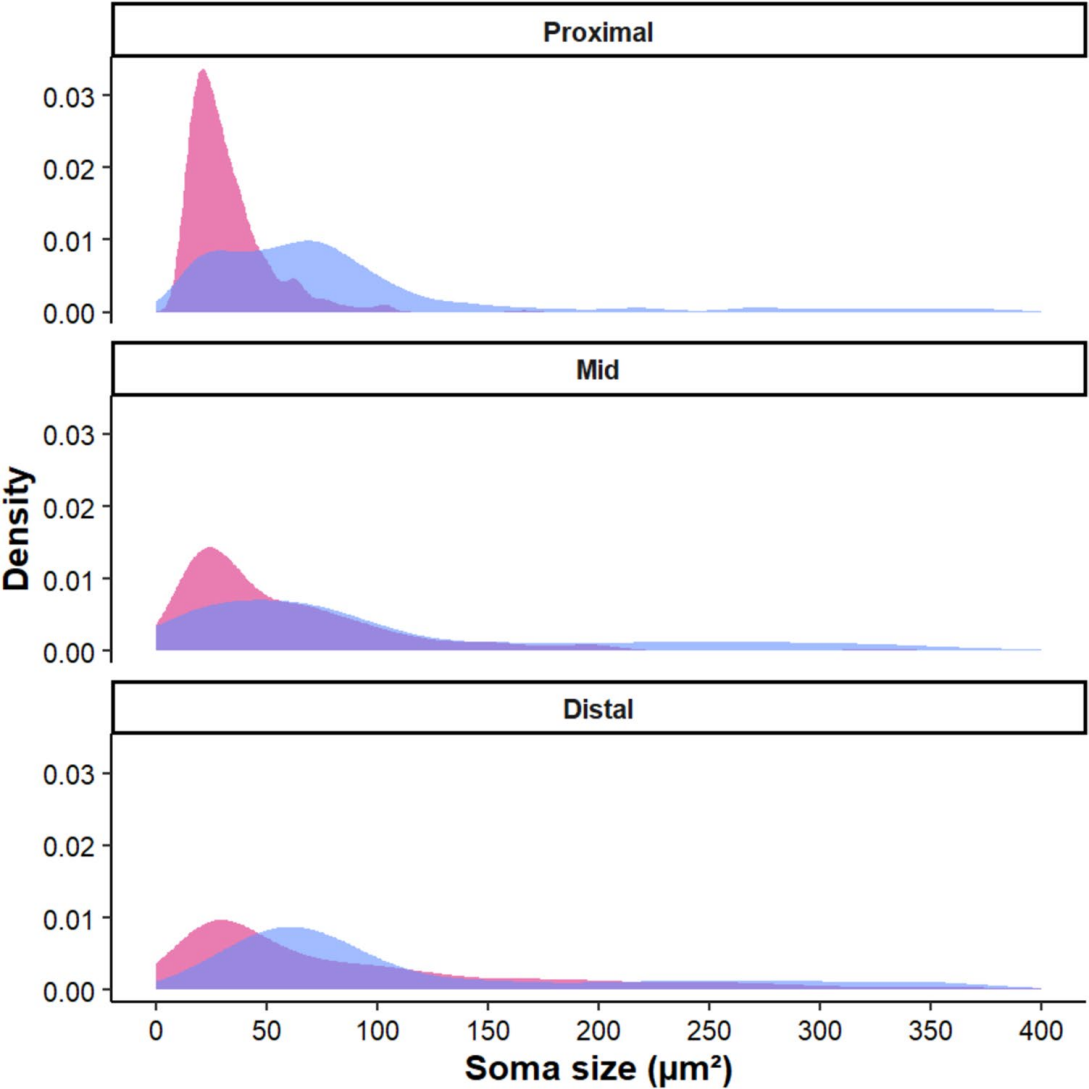
Density plots of neuronal soma size (μm^2^) within the myenteric plexus of barramundi (*Lates calcarifer*). Regional distributions are shown for the proximal, mid, and distal intestine. Cell bodies immunoreactive to the pan-neuronal marker Hu are represented in pink, and those additionally immunoreactive to nitric oxide synthase (NOS) in blue. NOS + neurons are generally larger than the average neuronal population (Hu +) and heterogeneity increases distally along the intestine

The distribution of soma sizes varied by neurochemical content and region (Table 2). Nerve cell bodies labelled exclusively with Hu were predominantly < 50 μm^2^ across all regions. Larger (> 100 μm^2^) non-nitrergic somata were more frequent and reached greater size in the mid and distal intestine. Nitrergic neurons showed a more varied size distribution but were consistently larger than the general population (Fig. 6). Two subpopulations of nitrergic neurons were distinguishable by a bimodal distribution of soma size in the barramundi proximal intestine (Fig. 6). The most abundant nitrergic neurons were between 50 and 100 μm^2^ in all regions. Like the general population, larger (> 100 μm^2^) nitrergic neurons were more common and reached greater sizes as the intestine progressed distally (Table 2).

**Table 2:** Average size of myenteric neurons labelled by pan neuronal marker Hu and co-labelled for nitric oxide synthase (NOS), in three regions (proximal, mid, and distal) of the barramundi (*Lates calcarifer*) intestine

	**Region**		
*Soma Size* (μm^2^)	***Proximal***	***Mid***	***Distal***
*Hu* +			
< *50*	***26.6* ± 9.62** **(*n* = 477)**	***26.3* ± 10.8** **(*n* = 206)**	***28.0* ± 9.34** **(*n* = 313)**
*50–100*	***64.7* ± 11.9** **(*n* = 67)**	***73.3* ± 13.8** **(*n* = 99)**	***72.4* ± 14.9** **(*n* = 136)**
> *100*	***114* ± 25.5** **(*n* = 6)**	***160.4* ± 58.2** **(*n* = 55)**	***195.0* ± 77.5** **(*n* = 222)**
NOS +			
< *50*	***29.5* ± 11.3** **(*n* = 203)**	***25.5* ± 11.7** **(*n* = 179)**	**34.8 ± 9.95** **(*n* = 139)**
*50–100*	***73.0* ± 13.1** **(*n* = 254)**	***72.3* ± 13.9** **(*n* = 182)**	**71.0 ± 13.4** **(*n* = 275)**
> *100*	***191.0* ± 11.3** **(*n* = 127)**	**228.3 ± 79.5** **(*n* = 166)**	***231.1* ± 86.5** **(*n* = 229)**

### Intestinal motility

Contraction of the smooth muscle cells in the muscularis externa is the foundation of intestinal motility. Video imaging of intestinal wall movements was used to monitor dynamic changes in contractility of the intestine of freshwater barramundi (*n* = 15). These recordings were transformed into diameter maps (Dmaps). From these maps, multiple distinct types of intestinal motility patterns could be described. Two patterns occurred frequently and consistently enough to allow for quantitative analysis, referred to here as: ‘proximal contractions’ and ‘longitudinal contractions’. Two other patterns: ‘shuttling’ and ‘mixing’, were invoked during the distended treatment. Lastly, a temporary but consistent change in behaviour was observed in all fish (*n* = 15) immediately after exposure to hexamethonium, referred to here as the ‘hexamethonium response’.

### Proximal contractions

**Fig. 7:**
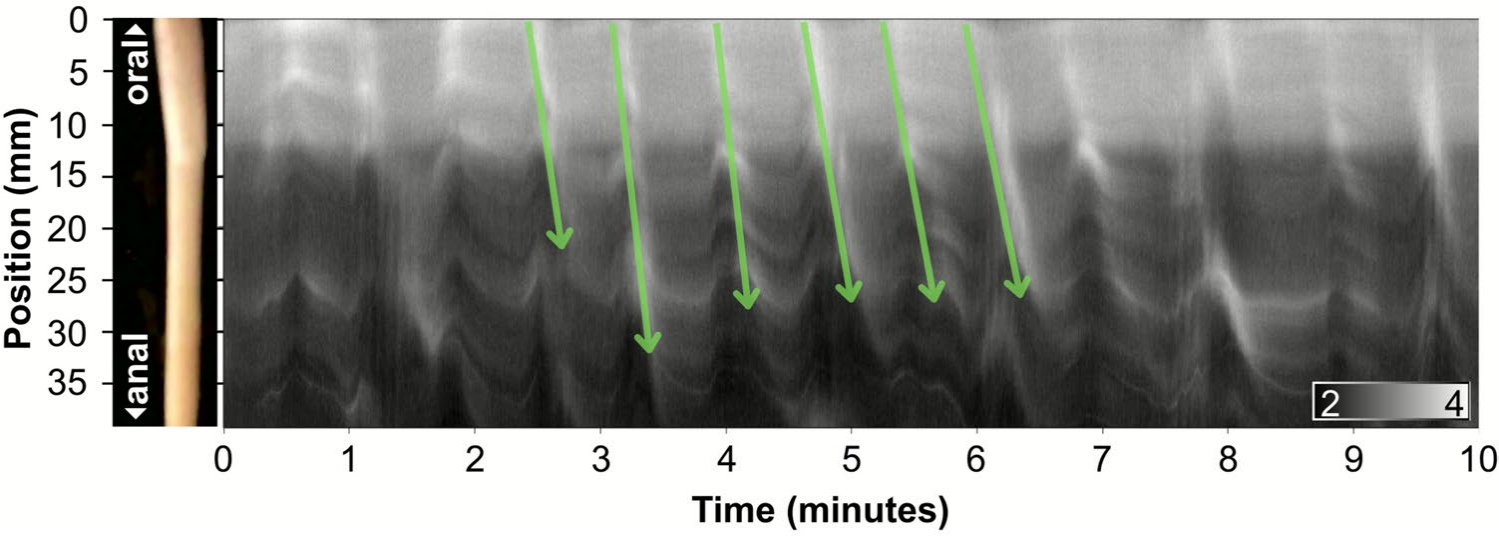
Diameter map (Dmap) showing proximal contractions (PCs) occurring in the oral-most 40 mm of the barramundi intestine. Green arrows indicate 6 examples of PCs that occur over a 10 min period and show the direction of travel. A raw image of the intestinal region recorded is provided on the left. The gradient in the bottom right corner represents the intestinal diameter of each point along the tract at any given time, where a completely black pixel corresponds to a diameter of 2 mm and a completely white pixel corresponds to a diameter of 4 mm

Proximal contractions (PCs) were short, antegrade, rhythmic contractions; unique to the proximal intestine (Fig. 7). PCs always initiated from the most oral point of the intestine and were consistently observed in all fish sampled (*n* = 15). Additionally, they were observed at least once, across all treatments.

Proximal contractions occurred roughly every 40 s, with a median frequency of 1.5 contractions per min (IQR: 1.1–1.7 cpm) and travelled a median distance of 10.7% (IQR: 7–12.1%) of the total intestinal length. They always propagated in an antegrade direction and overall had a median velocity of 1.16 mm·s^−1^ (0.69 −1.99 mm·s^−1^).

**Fig. 8:**
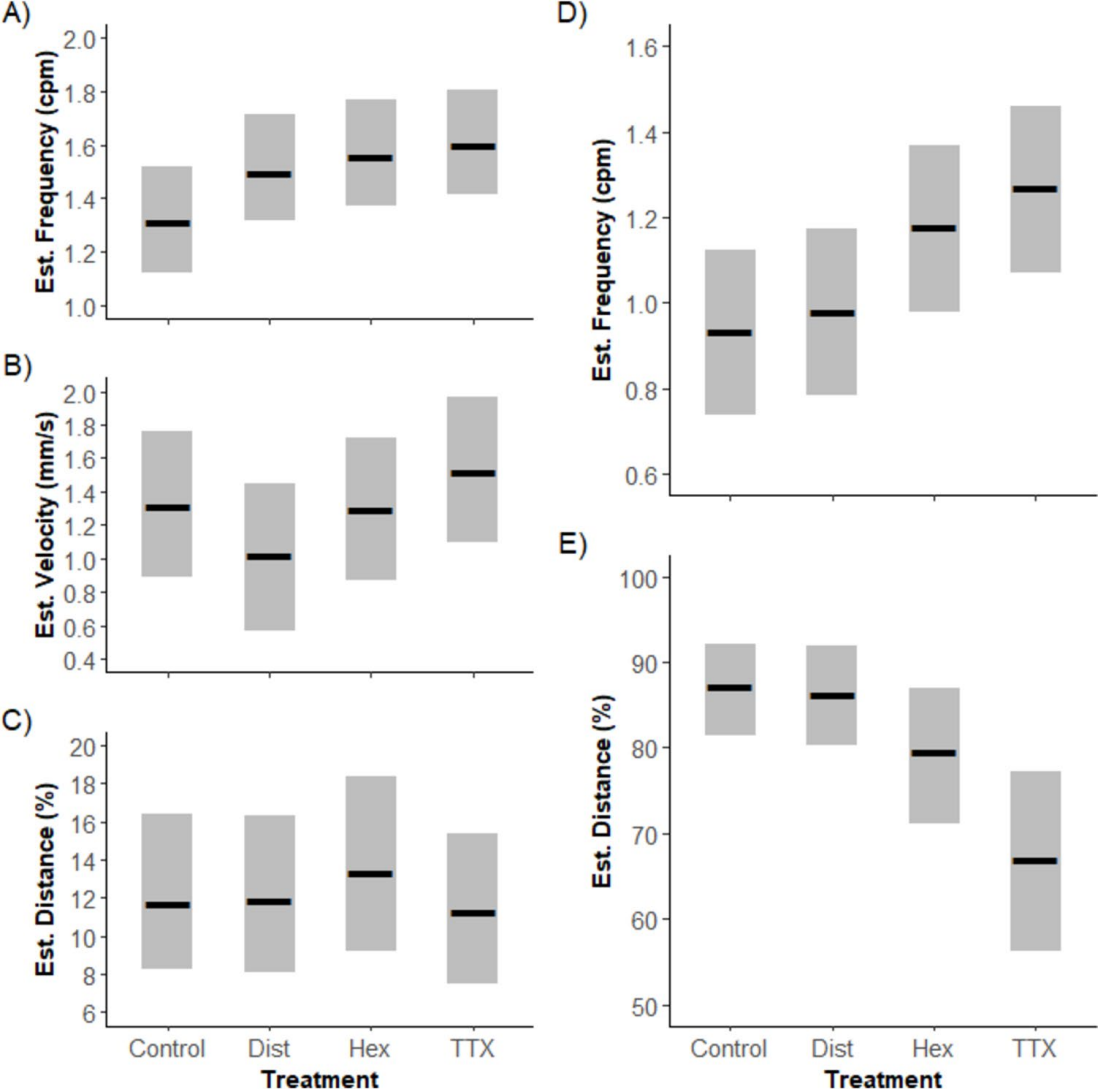
Estimated marginal means (EMMs), derived from posterior predications of proximal (A–C) and longitudinal (E–F) contractions in response to treatment (Control, Dist: distention, Hex: hexamethonium, TTX: tetrodotoxin). A, D Contraction frequency (cpm), B velocity (mm·s^−1^), and C, E distance travelled (percentage of total intestinal length). Black bars represent the estimated EMMs from the performed Bayesian mixed models, and bars show their corresponding uncertainty (95% credible intervals)

Distention activates mechanically sensitive ion channels in the ENS, leading to changes in motility. Distention increased the frequency of PCs by 0.19 cpm (CI: 0.12, 0.25) and decreased velocity by 0.30 mm·s^−1^ (CI: − 0.41, − 0.29), but had no meaningful effect on the extent of the pattern (distance travelled) (Fig. 8).

After exposure to hexamethonium, PCs persisted, increasing further in frequency (by 0.24 cpm compared to control; CI: 0.19, 0.30) and propagating 15% further (CI: 5, 24), whilst velocity was subtly affected but not deemed meaningful (− 0.03 mm·s^−1^, CI: − 0.12, 0.07). TTX continued to amplify PC’s, causing further increases in frequency and velocity, by 0.28 cpm (CI: 0.22, 0.35) and 0.20 mm·s^−1^ (CI: 0.09, 0.32) respectively, with no meaningful effect on propagation distance.

### Longitudinal contractions

Longitudinal contractions (LCs) were the most frequently observed motor pattern in the intestine. Observed in every sampled fish and across every treatment, LCs were characterised by rhythmic, transient reductions of the intestinal length. Whilst LCs were active in the proximal, mid, and distal intestinal regions, a consistent origin point of the contractions could not be resolved.

Under control conditions, LCs had a median frequency of 0.90 cpm (IQR: 0.70–1.30), approximately equating to one contraction every 67 s. The distance covered by LCs refers to the proportion of intestine displaced by the contraction, with a median average proportion of 89% (IQR: 77%–98%), or 89% of the total intestinal length (Fig. 8).

Unlike PCs, the frequency and distance of LCs were unaffected by distension. However, LCs persisted after HEX exposure and experienced a meaningful increase in frequency of 0.24 cpm compared to the control (CI: 0.15, 0.33). Likewise, LCs persisted in TTX, increasing in frequency by 0.31 cpm (CI: 0.04, 0.34) and decreasing in extent from control conditions by 20%, travelling along a predicted value of 67% of the intestinal length (C1: 63.2, 71.3).

Together, the data suggests LCs are a myogenic (non-neural), rhythmic contractile pattern that can occur in all regions of the barramundi intestine. Compared to PCs, LCs were insensitive to mechanical distension but again enhanced in frequency after exposure to HEX and TTX and therefore may also be under inhibitory neural control. PCs often occurred simultaneously with LCs, possibly suggesting a common origin. However, their differential responses to mechanical stimulation and examples of independent occurrence reiterate that PCs and LCs are independent behaviours.

### Shuttling and mixing

**Fig. 9:**
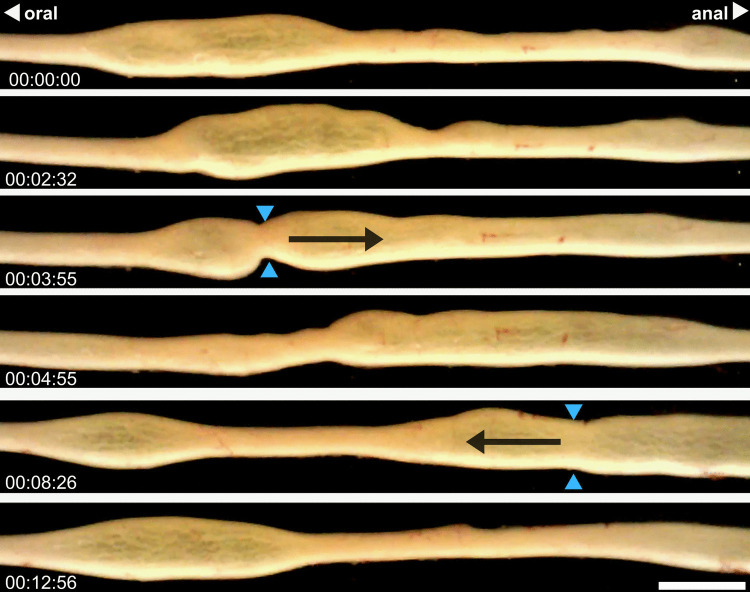
Progression of the distension-evoked myogenic pattern ‘shuttling’, observed in the whole intestine of a freshwater barramundi (*Lates calcarifer*). Time stamps showing the progression of the pattern are provided in the bottom left corner of each frame (hh:mm:ss). Blue arrowheads indicate points of localised circular muscle contraction and black arrows demonstrate the subsequent direction of travel of luminal content. Scale bars: 10 mm

Shuttling refers to a less common but prominent oscillatory pattern that was observed in the mid and distal intestine. This behaviour was not present in control conditions but emerged following distention. Shuttling typically began with a localised dilation near the oral end of the mid intestine (Fig. 9). Over ~ 5 min, the dilated region was propelled in an antegrade direction. Upon reaching the distal end, the contents were siphoned back to their original position, restarting the cycle. Hexamethonium temporarily disturbed the pattern, but motility resumed in the antagonist. Similarly, tetrodotoxin did not abolish shuttling but reduced the distance over which the pattern occurred. This pattern of activity appeared to be a distension-evoked myogenic pattern of the mid and distal barramundi intestine that is capable of antegrade propulsion.

Mixing refers to short, rapid, bidirectional propagations observed in the mid and distal intestine that emerged only after distention (*n* = 5 of 15). These contractions resembled ‘ripples’, a rhythmic, myogenic pattern previously described in small mammals. However, the contractions observed in barramundi were abolished within 10 min of TTX exposure, suggesting that, unlike ripples, mixing was dependent on neuronal activity.

#### The hexamethonium response

**Fig. 10:**
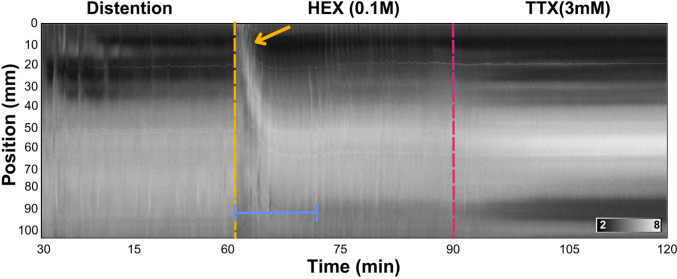
Diameter map (Dmap) demonstrating changes in barramundi (Lates calcarifer) intestinal width over time. Three treatments are presented: Distention, HEX: hexamethonium, and TTX: tetrodotoxin, all 30 min in duration. Partitioned lines indicate the point in time in which HEX (yellow) and TTX (pink) were added to the bath. The hexamethonium response was a sustained longitudinal contraction (yellow arrow) that slowly releases over the course of 13 min (blue range). In the gradient in the bottom right, black corresponds to a minimum intestinal diameter of 2 mm and white corresponds to a maximum diameter of 8 mm

A striking and consistent feature of each experiment was a prompt contraction response induced after the application of hexamethonium (HEX). These hex-evoked contractions propagated along the intestine. The antagonist also promptly induced a prolonged and intense LC that would originate from the most oral point of the intestine. This contraction was sustained for 2–3 min before a slow release, after which a series of 5–6 smaller LCs occurred and slowly progressed in an antegrade direction along the tract over the course of 10–15 min (Fig. 10). Luminal contents would subsequently be propelled to the mid and distal intestine, where they would be held indefinitely. Ongoing motor patterns were temporarily disrupted by the hexamethonium response but reached a new, stabilised baseline after 15 min. This acute contractile response to the application of hexamethonium is compatible with the possibility that the enteric neurons of the barramundi intestine use nicotinic transmission and exert an ongoing inhibitory influence on the smooth muscle.

## Discussion

This study provides the first description of enteric neurons and gut motility in isolated barramundi intestine. Anatomically, the barramundi GI tract follows generalised patterns described for teleost fish (Ray and Ringø 2014), and our descriptions agree with those of Purushothaman and colleagues (Purushothaman et al. 2016), who previously described barramundi GI morphology in great detail. Barramundi show a high level of compartmentalisation between gut regions for a teleost (Ray and Ringø 2014); and the species’ prominent y-shaped stomach and short intestines (RIL < 1) align with its’ carnivorous diet (Buddington et al. 1997; Duque-Correa et al. 2024; Purushothaman et al. 2016). We report that the barramundi intestine can be further subdivided into a proximal, mid, and distal region. There is ambiguity in the literature regarding whether the mid-intestinal region is a distinct anatomical region or just a transitionary extension between two more well-defined regions in teleosts (Abad et al. 1987; Buddington et al. 1997). Here we use differences in tissue structure, motility between regions, and the prominence of the two flexures that fold the intestine into three zones in vivo to support that the mid intestine is its own region, different in structure and possibly function.

The muscularis externa of the barramundi intestine consists of two distinct smooth muscle layers that lay perpendicular to each other: an inner circular layer and an outer longitudinal layer. These layers vary in thickness between regions and circumferentially. The thickness of the circular muscle was greatest in the proximal and distal intestine, with a notable reduction in the mid intestine. This variation likely coincides with differences in functional demand across regions. Between the smooth muscle layers in every region, there is a distinctive band of connective tissue. Histochemical reports in salmonids label a comparable tissue to the myenteric plexus (Løkka et al. 2013). This was confirmed as such in the barramundi through immunohistochemical staining, where all identified nerve cell bodies were solely contained within a myenteric plexus at the same location. There was no evidence of a submucosal plexus, consistent with reports in other teleosts (Olsson 2009). The significance of this absence remains unclear. In mammals, the two major plexuses specialise in different functions; the submucous plexus is necessary for regulating mucosal functions (e.g., transepithelial ionic secretion and absorption) (Keast 1987; Frieling et al. 1992; Weber et al. 2001), while the myenteric plexus is required for gut motor coordination (Costa and Furness 1976). Thus, it is possible these functions are controlled from a single plexus in the barramundi and other teleosts, and/or that extrinsic nerve fibres play a relatively large role in regulating mucosal functions.

The spatial organisation of nerve cell bodies in the barramundi myenteric plexus is sparse and unganglionated, which is consistent in all regions. Our descriptions of the myenteric plexus labelled with HuC/D are similar to other teleosts, including zebrafish (Kuil et al. 2021; Olsson and Holmgren 2011), gilted sea bream (*Sparus aurata*) (Ceccotti et al. 2018), shorthorn sculpin (Olsson 2009), and brown trout (*Salmo trutta*) (Burnstock 1959). Despite a lack of ganglia, enteric nerve cell bodies in barramundi are well connected by nerve fibres, thus satisfying the definition of a plexus (Sharkey and Mawe 2023). Notably, these descriptions deviate substantially from those given for mammals, including rabbit (*Oryctolagus cuniculus*) (Kigata et al. 2025) and guinea pig (*Cavia porcellus*) (Steele et al. 1991), that exhibit prominent ganglia.

Interestingly, structural variation of the myenteric plexus shows between-species variation among teleosts. Whole-mount preparations presented in Olsson’s 2009 review (Olsson 2009) of autonomic innervation in the fish GI tract offer visual evidence towards these differences. In zebrafish, the myenteric plexus is strikingly simple in structure; nerve fibres are thin, sparse, and oriented longitudinally (Olsson 2009; Uyttebroek et al. 2010). Conversely, the shorthorn sculpin possesses a more complex network, exhibiting a greater number of interconnections between nerve cell bodies (Olsson 2009, 2011a, b). The structure of the barramundi myenteric plexus appears intermediate between these two patterns. Most fibres are oriented longitudinally, like that of zebrafish (Olsson 2009), but are notably thicker and display more interconnections, as in the shorthorn sculpin (Olsson 2009).

We labelled two populations of neurons in the myenteric plexus of the barramundi: one immunoreactive to NOS, the other non-nitrergic. Like in other vertebrates, a substantial percentage of myenteric neurons expressed nitric oxide (NO) and presumably play a key role in neuromuscular inhibition. Indeed, NOS immunoreactive fibres were abundant in the circular muscle layer, suggesting that inhibitory motor neurons to the circular muscle are among the NOS immunoreactive myenteric nerve cell bodies observed in this study. Nitrergic neurons had a larger soma size, compared to the general Hu + neuronal population, suggesting the subpopulation represents a morphologically distinct class within the myenteric plexus. This is consistent with other vertebrates, where inhibitory motor neurons that express NO are often characterised by larger somata. Heterogeneity of soma size increased distally along the intestine and appeared most uniform in the distal region and likely reflects a change in function along the organ.

The highest proportion of nitrergic neurons was found in the proximal intestine, with a progressive decrease toward the distal end. This contrasts with the proportion of nitrergic neurons found in the intestine of zebrafish (Uyttebroek et al. 2010), rainbow trout (*Salmo gairdneri*) (Li and Furness 1993), and masu salmon (*Oncorhynchus masou*) (Pimenova and Varaksin 2012). Zebrafish exhibit a progressive increase of nitrergic neurons in the intestine as juveniles (Uyttebroek et al. 2010), but are a consistent proportion along the intestine as adults (Uyttebroek et al. 2010). Brown trout exhibits a marked increase in nitrergic neurons abundance in the distal intestine (Li and Furness 1993). In the masu salmon, the number of nitrergic neurons is greatest in the mid intestine, with a slight decrease in the proximal and distal regions (Pimenova and Varaksin 2012). While an interesting observation, the implications of this diversity between species remain unclear as barramundi, masu salmon, and brown trout are all carnivorous, euryhaline, and of similar evolutionary complexity.

Labelling for the neuropeptide calcitonin gene-related peptide (CGRP) returned a positive response in nerve fibres only. A similar finding was noted in zebrafish, with or without colchicine pretreatment (Uyttebroek et al. 2010). The absence of CGRP-labelled nerve cell bodies could have multiple explanations. One possibility is that the labelled fibres represent extrinsic primary afferent inputs rather than intrinsic enteric neurons. However, technical limitations in antibody sensitivity may have also prevented the detection of CGRP-expressing enteric neurons. CGRP-positive enteric cell bodies have been labelled previously in the gilthead sea bream (Ceccotti et al. 2018). As such, while a positive response to fibre labelling is confirmation of some form of interconnective network, the negative response to labelling nerve cell bodies in barramundi does not indicate their absence (Burkhardt-Holm and Holmgren 1989). Such limitations are still a common challenge in studying the teleost ENS with mammalian raised antibodies as species-specific responses are highly variable (Burkhardt-Holm and Holmgren 1989; Olsson 2009).

### Autonomic control of intestinal motility in barramundi

In the barramundi, four intestinal motility patterns were commonly observed: proximal contractions (PCs), longitudinal contractions (LCs), shuffling, and mixing. In mammals, intestinal motility is tightly modulated by neuronal pathways of the ENS. This appears to hold true in the barramundi as well. We showed that mechanical distention and antagonists of neuronal transmission or nerve conduction altered frequency, velocity, and distance of intestinal motility patterns in the barramundi.

Mechanical distention effected each intestinal motility pattern differently. The frequency of PCs increased, but the frequency of LCs was unaffected. Additionally, shuffling and mixing, though less frequent, only initiated *after* distention and thus may be dependent on the presence of an ongoing mechanical stimulus. The change in intestinal motility behaviour after distention is suggestive of the presence of mechanosensitive enteric neurons (MEN) (Smith et al. 2007; Spencer and Hu 2020). Intrinsic mechanosensory neurons respond to the compression or stretching of the GI tract walls, subsequently initiating reflex activity, transmitting signals from sensory neurons to the smooth muscle layers and/or motor neurons (Smith et al. 2007; Spencer and Smith 2004). Mechanosensitive enteric neurons have been well described in mammals, and an analogous response attributed to MEN has been noted in the brown trout (*Salmo trutta) (*Burnstock 1958, 1959*)*. However, the mechanisms and dependencies of MEN in intestinal motility in teleosts have yet to be formally explored.

### Hexamethonium and tetrodotoxin

Intrinsic GI motility can be altered and observed through the addition of specific neurotransmitters or drugs that target nicotinic and adrenergic receptors. This is the basis for many early in vitro studies of GI motility in all vertebrates and to date still constitutes most of the work done in teleosts.

Exposure to the ganglionic blocker hexamethonium (HEX) consistently evoked an episodic increase in the frequency and velocity of intestinal motility in barramundi. We termed the distinctive change in behaviour, which would resolve after 15 min, the ‘hexamethonium response’. This response involved a maintained longitudinal muscle contraction that propagated aborally across the proximal and mid regions, diminishing before it reached the distal intestine. Upon completion of these contractions, PCs and LCs maintained an increased frequency. This implicates nicotinic receptors in an ongoing inhibitory mechanism, whose suppressing effect on PCs and LCs is released on HEX application.

The application of hexamethonium appears to evoke a variation of responses in the teleost GI tract. In brown trout, exposure to HEX abolishes peristaltic activity in the intestine (Burnstock 1958, 1959). Such blockade was not observed in the intestine of barramundi, nor does it appear to occur in silver perch (*Bidyanus bidyanus*) (Jones et al. 2021). What was cohesive between species after HEX exposure was an increase in the frequency of longitudinal contractions over a 20-min period, which was similarly described independently in all three species (Burnstock 1958; Burnstock 1969; Jones et al. 2021). It is unclear whether this variation in the alteration of peristaltic activity is a species-specific response or affected by differences in application technique and location between studies.

The sodium channel blocker, tetrodotoxin (TTX) abolished the mixing-type motor pattern in barramundi, indicating a neuronal dependence for this behaviour and further functional evidence compatible with the neuroanatomical identification of enteric neurons. While other intestinal motility patterns persisted in TTX (PCs, LCs, and shuttling), their frequency, velocity, and distance were fundamentally changed after exposure. This suggests these behaviours are myogenic but may be subject to modification by neuronal activity. The change in each parameter was always consistent in direction with the pattern’s prior response to HEX. For example, the frequency and velocity of PCs were further increased by TTX, suggesting a component of neuronal influence over this behaviour does not require nicotinic transmission.

These results provide the first description of the ENS in barramundi and demonstrate a role for the ENS in the modulation of GI motility ex vivo. The structure of the myenteric plexus in barramundi is comparable to other teleosts but deviates somewhat from the common mammalian model. In teleosts, the organisation of the ENS differs from terrestrial mammals; despite performing a complex range of functions, including osmoregulation. An increased proportion of inhibitory neurons in the proximal intestine suggests that regulatory behaviours in teleosts are potentially related to the diet of the species.

## Data Availability

The authors confirm that the data supporting the findings of this study are available within the article and or its supplementary materials.
